# Re-annotation of the *Saccharopolyspora erythraea* genome using a systems biology approach

**DOI:** 10.1186/1471-2164-14-699

**Published:** 2013-10-11

**Authors:** Esteban Marcellin, Cuauhtemoc Licona-Cassani, Tim R Mercer, Robin W Palfreyman, Lars K Nielsen

**Affiliations:** 1 Australian Institute for Bioengineering and Nanotechnology (AIBN), The University of Queensland, Brisbane, Qld 4072, Australia

**Keywords:** Proteogenomics, *Saccharopolyspora erythraea*, Systems biology, Genome annotation, High G + C content genomes

## Abstract

**Background:**

Accurate bacterial genome annotations provide a framework to understanding cellular functions, behavior and pathogenicity and are essential for metabolic engineering. Annotations based only on *in silico* predictions are inaccurate, particularly for large, high G + C content genomes due to the lack of similarities in gene length and gene organization to model organisms.

**Results:**

Here we describe a 2D systems biology driven re-annotation of the *Saccharopolyspora erythraea* genome using proteogenomics, a genome-scale metabolic reconstruction, RNA-sequencing and small-RNA-sequencing. We observed transcription of more than 300 intergenic regions, detected 59 peptides in intergenic regions, confirmed 164 open reading frames previously annotated as hypothetical proteins and reassigned function to open reading frames using the genome-scale metabolic reconstruction. Finally, we present a novel way of mapping ribosomal binding sites across the genome by sequencing small RNAs.

**Conclusions:**

The work presented here describes a novel framework for annotation of the *Saccharopolyspora erythraea* genome. Based on experimental observations, the 2D annotation framework greatly reduces errors that are commonly made when annotating large-high G + C content genomes using computational prediction algorithms.

## Background

Genome annotations are essential to study and manipulate microorganisms. With advances in next generation sequencing, genomes are released with ever increasing frequency and with them, new annotation pipelines are emerging [1-3]. Most pipelines rely entirely on *in silico* prediction tools, and therefore, fail to accurately determine gene start/stop and to precisely assign gene function [4-6]. For example, Nielsen *et al.* found that 60% of the annotated bacterial genomes contain substantial errors in start/stop codons predictions and are generally over-annotated due to a lack of thorough analysis between computationally assigned open reading frames (ORFs) and real genes [7]. This observation has been acknowledge by the National Centre for Biotechnology Information (NCBI), which is constantly developing their Prokaryotic Genome Automatic Annotation Pipeline (PGAAP) since 2003 [8]. Additionally, NCBI also routinely runs Glimmer, GeneMark and Prodigal on all complete genomes and plasmids, and makes the results available in the FTP directory of each organism.

Errors in annotation are particularly abundant in large, high G + C content genomes, where gene length and gene organization vary significantly from well-annotated model organisms such as *Escherichia coli*, *Saccharomyces cerevisiae* or *Bacillus subtilis*. In fact, Prodigal was developed after it was observed that the accuracy in gene recognition drops considerably for high G + C content genomes [9]. G + C rich genomes have considerably fewer overall stop codons and larger numbers of spurious open reading frames (ORFs). A comparison of Genebank genomes and prodigal genome annotation showed that false ORFs are often selected instead of the real ORFs within the same genomic region [9]. These long ORFs also contain a large numbers of potential start codons that lead to a considerable drop in accuracy of the translation initiation site prediction and tend to predict too many genes [9].

Advances in *omics* offer new opportunities to perform functional genome annotations. Recently, Qiu *et al*. [10], performed a functional 2D annotation of the *Geobacter sulfurreducens* genome. Integration of proteomics, transcriptomics and Chip-seq enabled the precise re-annotation of the genome. Similarly, other authors have used proteogenomics to provide an unbiased but direct correlation between genome sequence and protein expression [11-14]. Annotation of 46 bacterial and archea genomes using this approach has shown that purely bioinformatics-based pipelines fail to annotate mainly short-length proteins and high G + C content sequences [15].

High G + C content genomes encompass the majority of *actinobacteriaea*, a distinct bacterial phylum capable of producing numerous antibiotics [16]. They include soil and marine industrial microorganisms as well as numerous animal and human pathogens. Due to their relevance, a large number of actinomycete genomes have been sequenced. *Saccharopolyspora erythraea (S. erythraea)* is an important industrial antibiotic producer, a model actinomycetes and one of the first actinomycetes genomes sequenced. Its circular genome was sequenced in 2007 [17], and comprises 8.2 Mbp (72% G + C) with the potential to synthesise more than 25 bioactive secondary metabolites [17]. Similar to most actinomycetes, *S. erythraea’s* annotation was based on sequence homology and hence, prone to the associated annotation inaccuracies.

The accurate annotation of the *S. erythraea* genome is of significant importance, not only for its biotechnological significance but also as a model to functionally annotate other G + C rich genomes. Here, we propose a functional 2D re-annotation of the *S. erythraea* genome by combining *in silico* predictions with a multi-*omics* approach. By integrating proteomics, transcriptomics and the use of a genome-scale metabolic reconstruction (GSMR) we show the value of a systems biology driven annotation tool for the prediction of novel genes and accurately map ribosomal binding sites across the genome.

## Results

### Annotation of G + C rich genomes using Prodigal 2 and GenePRIMP improves genome annotation

In an effort to perform a whole genome re-annotation using Prodigal 2 [9], ORFs were annotated using BLASP. Genes found by prodigal were BLASTed against related high G + C content genomes separately and against the National Centre for Biotechnology Information (NCBI) nb database, Interpro and SwissProt databases for missing domains. InterProScan was used to assign Go/InterPro IDs to hypothetical proteins. The tRNAs were identified using tRNAscan-SE and rRNAs were identified using rRNA_hmm_fs. In total, 7,454 coding sequences were found: 50 tRNAs, 125 rRNAs and 7,279 genes. This re-annotation represents an increase of 78 genes compared to the previous annotation. Of all ORFs found by prodigal, only 2,183 genes had an EC number associated to specific genes. To further explore the coding potential of the genes, all genes predicted as hypothetical proteins (> 45% of the total coding sequences) were assigned to a GO term or an Interpro ID using InterProScan. Using this approach, we assigned GO/InterPro IDs to 2,015 out of 3,330 hypothetical proteins (1,119 hits with unique GO term assigned).

To further resolve errors in annotation, the novel and previous annotations were submitted to the Gene PRediction IMprovement Pipeline (genePRIMP) [6]. The new annotation is available as Additional file 1. Despite numerous errors in both annotations, Prodigal 2 predicted 46 less anomalies than the previous pipeline (Total anomalies = 1,017 and 971 respectively). The use of GenePRIMP for genome annotation enabled manual revision of 132 long genes, 130 short genes, 99 broken genes and 76 interrupted genes. Additionally, 560 putative missed genes, identified from the alignment of a gene or intergenic region to its homologs were detected.

### RNA sequencing revealed transcription of more than 300 intergenic regions

**Figure 1:**
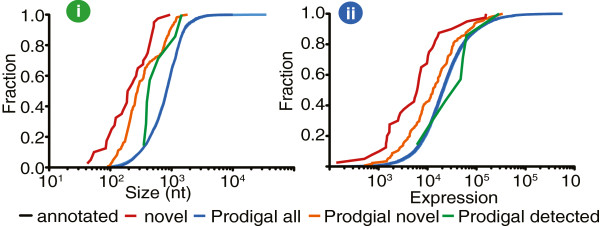
Cumulative frequency distribution showing the (i) relative size and (ii) expression for annotated, novel and genes detected by prodigal relative to previously genome annotation.

A close investigation into the recently published RNA-sequencing data from *S. erythraea*[18], revealed substantial transcriptional content (32%) originated from un-annotated sequences. Analysis of such regions revealed transcription of ~300 intergenic regions that displayed intergenic transcription (Additional file 1: Table S2, Figure 1), which largely coincide with the anomalies indicated by Gene PRIMP. Previously, 190 of these intergenic regions were annotated as potential ncRNAs using a range of metrics, including presence, size and structure to annotate novel independent ncRNAs within intergenic regions [18]. The novel ncRNAs displayed distinct CPC scores [19], dynamic transcriptional pattern and 14 of them showed a distinct ncRNA secondary structure. Furthermore, these regions displayed strong similarities to genes from related microorganisms (Additional file 1: Table S2).

### Proteogenomics was used to validate novel ORFs

**Figure 2:**
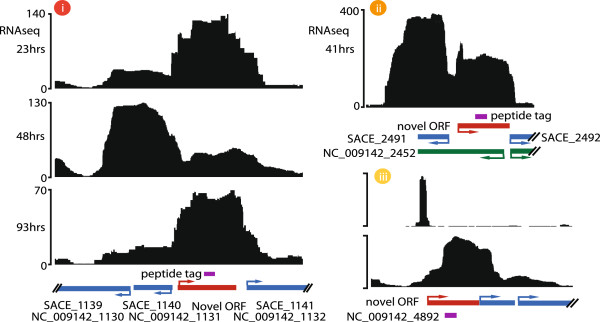
**Proteogenomics approach for novel protein annotation.** Examples of novel open reading frames (ORFs) detected by proteogenomics. ORFs (red) were detected by the in-frame expansion of peptide spectra (purple) that uniquely map to un-annotated intergenic regions. The RNA sequencing coverage profile from various sample time points (black histogram) associated with these examples is also indicated. **(i)** Expression of these novel proteins displaying a dynamic transcriptional profile. **(ii)** Validation of novel ORF found with Prodigal 2. **(iii)** A small RNA associated with the initiation codon.

To validate the coding potential of the novel annotated regions, we combined transcriptional data with 2D-LC MS/MS proteomics. A total of 1,139 distinct proteins were identified from 6 different fermentation time points (Figure 2, Additional file 1: Table S3). With this approach, 164 ORFs, previously annotated as hypothetical proteins were confirmed (Additional file 1: Table S4). More importantly, the alignment of peptides to the intergenic regions confirmed the expression of 58 previously unidentified proteins (Additional file 1: Table S5). Using proteogenomics we validated several of the novel genes found by prodigal 2 (Figure 2 ii). For example, a peptide was found in the intergenic region between SACE_2491 and SACE_2492, which was found to be contained in the gene NC_009142_2452. The peptide GDNAVLALVESAGNSGPNLRASKLR, translated 2 bp from SACE_1312/NC_009142_1296 (in the same coding frame), potentially represents a miss-annotation error. As evidenced previously [4], incorrect annotation of the ORF is caused by a frame shift in the sequence presumably due to a missing base in the genome sequencing. Neither Prodigal nor FrameD [20] were able to find a new stop codon upstream of the peptide in the same frame. All novel proteins with a single peptide match were manually validated with their RNA-seq transcriptional profile (Figure 2). Only after analysing peptides in terms of gene proximity and frame localisation, was the correct annotation of 13 ORFs and the re-annotation of 44 distinct full-length proteins achieved.

### Small RNA-sequencing enabled ribosomal binding sites (RBS) annotation

**Figure 3:**
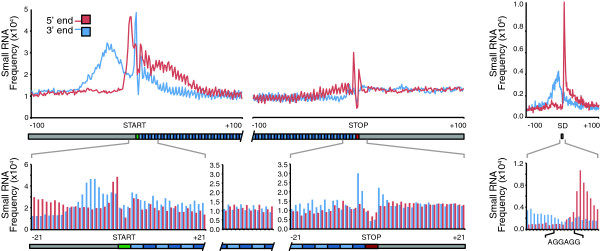
**Annotation of TSS using small RNA sequencing.** Frequency distribution of RNA fragment 5′ (red) and 3′ (blue) termini aligning sense to mRNA strand. We observed protection of the initiation (green box) with 3 nt periodicity to the stop codon (red) box. Distribution of RNA fragments 5′ (red) and 3′ (blue) to predict transcription start sites (TSS) for genome annotation. Top panel indicates the protection of the Shine-Dalgarno (SD) sequence.

We have previously used deep-RNA sequencing to demonstrate targeted mRNA degradation during the growth arrest stage (metabolic switch) in the *S. erythraea* developmental cycle [18]. In this work, the mRNA degradation event was used to resolve ribosomal binding sites. A detailed inspection of the alignment profile of small RNAs during the metabolic switch, showed that the most abundant fragment reads for a given transcript coincided with positions where the ribosomes stall (Shine-Dalgarno), thereby protecting the transcript from endogenous cleavage. This was validated by our proteogenomics analysis, which found new proteins that contained clear evidence of RBS (Figure 2 iii). Alignment of small RNA sequences strongly coincides with the Shine-Dalgarno sequence (Figure 3). We also observe a 3 bp periodicity in small RNA occurrence, coinciding with the transcription programme of the ribosome (Figure 3).

### Use of the genome-scale metabolic reconstruction (GSMR) to improve functional annotation

We recently reconstructed a metabolic network based on the genome of *S. erythraea*[21], which was used to direct amino acid media supplementation strategies to improve erythromycin production. The reported metabolic reconstruction consists of 1,482 reactions (2,075 genes) and 1,646 metabolites, from which, as part of the manual curation, 108 reactions were added and 10 were identified as essential for growth in minimal media. Here, we use GSMR *in silico* simulations to provide evidence for the presence of these orphan enzymes (Additional file 1: Table S6). As previously reported for the closely related organism *Streptomyces coelicolor*[22], we searched for ORFs that fulfil specific functions and validated our findings by searching for gene synteny with related actinomycetes.

A common example of missing gene annotation in most actinomycetes is for the enzyme cardiolipin synthase [22]. This enzyme contains two phospholipase D-like domains (PLDc) and catalyses the condensation of two phosphatidyl-glycerol molecules into cardiolipin. Screening of the *S. erythraea* genome revealed a strong candidate ORF containing two PLDc domains. Therefore, we suggest that SACE_4234/NC_009142_4185 might be performing such enzymatic activity. Similarly, UDP-glucose-D-galactose-1-phosphate-uridylyltransferase requires a GalP_UDP transferase domain at each end of the protein. The ORF SACE_0764/NC_009142_0762 emerged as the sole candidate to fulfil this metabolic function. Comparably, a pyrophosphatase protein domain was found in ORF SACE_0391/NC_009142_0390, implying its re-annotation as inorganic diphosphatase.

The next orphan enzyme identified by the GSMR was the enzyme phosphatidylethanolamine-N-methyltransferase, which contains a PEMT (phospholipid methyltransferase) domain. The genome of *S. erythraea* contains two ORFs with such domain (SACE_0625/NC_009142_0625 and SACE_6539/NC_009142_6415); however, these genes also contain other domains, including MFS_1 (Major Facilitator Superfamily) and PhaG_MnhG_YufB (Na+/H + Antiporter subunit). The multiple domains contained within such enzymes prevented us to clearly identify a candidate ORF to fulfil that particular enzymatic function. Similarly, the enzyme (*S*)-3-hydroxyisobutyryl-CoA hydrolase contains two domains, ECH and ECH_C (enoyl-CoA hydratase/isomerse family). The *S. erythraea* genome contains 27 ORFs with at least one of those domains and four ORFs contain both domains with high confidence (SACE_1458/NC_009142_1441, SACE_1464/NC_009142_1447, SACE_2740/NC_009142_2703 and SACE_5406/NC_009142_5338) (E value < 0.001). After analysing all neighbouring genes, we concluded that the most likely genes to fulfil such function were genes SACE_1458/NC_009142_1441 and SACE_1464/NC_009142_1447. Those two genes share synteny with members of the family of *Mycobacterium* and *Corynebacterium*. Similarly, three ORFs (SACE_6460/NC_009142_6335, SACE_6548/NC_009142_6424 and SACE_6779/NC_009142_6657) contain the PGM_PMM (phosphoglucomutase-phosphomannomutase) domains required to perform the reaction of N-acetyl-D-glucosamine-1-phosphate-1,6-phosphomutase. The three genes have similar genomic context; however, SACE_6779/NC_009142_6657 performs the same chemical reaction with a similar substrate (glucosamine 1-phosphate). It is possible that it has relaxed substrate specificity and binds N-acetyl glucosamine 6-phosphate in addition to N-glucosamine 6-phosphate.

Finally, our search to annotate the ATP deoxyuridine 5'-phosphotransferase, which contains a thymidine kinase (TK) domain, was unsuccessful. This result highlights the need for better genome annotations, especially for high G + C content microorganisms where the reaction might be performed by an unrelated gene or by promiscuous enzymes [23].

## Discussion

It is well accepted that Genebank files, particularly genomes annotated more than 10 years ago, contain many mistakes. This has been evidenced when comparing gene bank files with Prodigal gene predictions. However, despite these observations, most groups are still using these genebank files as the reference for *omics* comparison, generation of metabolic network reconstructions or for metabolic engineering. Current genome annotation pipelines predict gene function based on sequence homology. However, when there is insufficient similarity between the query and the database, gene function cannot be predicted. This problem is exacerbated for pseudo-genes, genes with programmed or artificial frame shifts or high G + C content genomes [6,7]. Out of the ~25 million ORFs (encompassing approximately 2,000 bacterial genomes) deposited in PATRIC, 6.7 million are categorised as hypothetical proteins [24]. This indicates that, on average, 30% of the bacterial coding potential remains unknown. In the *S. erythraea* genome, more than 45% of its ORFs are annotated as hypothetical proteins [17]. The large number of genes with unknown function is likely to be the result of random horizontal gene transfer, an unusually high G + C content and a large genome. Comparison between the current genebank file and the prodigal annotation for *S. erythraea,* evidenced that in addition to the 7,190 genes initially predicted, 89 new genes were found and 2,085 genes differed in the re- annotation of start sites. A close comparison between the previous annotation and the prodigal annotation revealed that 995 genes differed in gene start/end site (Additional file 1: Table S1).

A combination of *in silico* predictions, RNA-seq, proteomics and the use of a GSMR were used to improve the genome annotation of *S. erythraea*. Analysis of RNA-seq data identified ~300 intergenic regions with high expression [18]. Using the genePRIMP pipeline we found that most of these transcribed regions have extensive homology to un-annotated regions in other species, highlighting the importance of a new annotation. The use of the GSMR also enabled the identification of orphan enzymes required for growth in minimal media.

Proteogenomics further identified novel ORFs with robust and dynamic expression despite their notably smaller size (Figure 1). Discovery of these novel features not only demonstrates the value of proteogenomics to correct genome annotation errors, but also confirms the lack of sensitivity of gene prediction tools for annotating genomes. In fact, a proteogenomic data comparison between various bacteria and archea genomes found that the number of annotation errors increase for short-length high G + C content sequences [15].

Ribosomal binding site prediction is normally achieved by sequencing RNA after nuclease digestion and ribosomal recovery by ultracentrifugation [25,26]. A detailed protocol of the current method is described by Ingolia *et al.*[26]. The protocol has been used to map RBS in embryonic stem cells [27], to study the effect of drugs in cancer therapy [28], for mapping of the RBS in yeast [25] and to study translation dynamics in bacteria [29]. In actinomycetes, RNase and protease activities regulate the developmental cycle [30,31]. It has been recently demonstrated that endogenous nuclease activity (specifically RNase III) occurs -and is required- for antibiotic production and proper mycelia development in *S. coelicolor*[30]. In fact, during the metabolic switch in *S. erythraea*, the entire transcriptome is reorganised by a tightly regulated targeted mRNA degradation programme [18]. In this work, this endogenous RNase activity was used in analogy to the *in vitro* nuclease digestion from the ribosomal foot printing protocol. We found that this *in vivo* RNA degradation is suitable for RBS profiling in actinomycetes.

## Conclusion

Actinomycetes are able to produce a large number of secondary metabolites of great pharmaceutical and industrial importance. However, as shown here, large G + C-rich genomes require experimental validation for accurate genome annotation. The combined use of proteogenomics, mRNA sequencing and a genome-scale metabolic reconstruction greatly improved genome annotation. Better genome annotations are likely to disentangle the fascinating and largely unexplored, genome potential of actinomycetes.

## Methods

### Bacterial strain, growth and fermentation conditions

*S. erythraea* (NRRL2338) was grown in 2-L bioreactors (Applikon) in mineral medium MM-101 without casamino acids, as previously described [18]. Medium ISP 2 (yeast extract, 4 g/L; malt extract, 10 g/L; Dextrose, 4 g/L; Agar, 20 g/L) was used as solid media for spore germination and seed cultures. Approximately 0.5 mL of glycerol stock was used to inoculate a 500 mL baffled flask with 100 mL of ISP 2 media incubated at 30°C in a rotary shaker (INFORS HT, Bottmingen, Switzerland) at 220 rpm for 30 h. When the seed culture reached an OD_450_ of 2.5 (early stationary phase), a second seed culture (1 L baffled flasks with 150 mL of ISP 2) was inoculated to an initial OD_450_ of 0.3 and incubated under the same culture conditions for 72 h. Cells were then centrifuged at 10,000 rpm at room temperature (Allegra X-15R, Beckman Coulter, USA), washed and resuspended in MM-101 prior to inoculation. Temperature and pH remained constant at 30°C and 7.0 respectively. Dissolved oxygen was maintained between 45 and 60% of saturation by increasing the air flow and the reactor mixing. Oxygen uptake rate (OUR) and CO_2_ production were measured using a mass spectrometer (Hiden, England) attached to the bioreactor’s condensers. Cells were harvested from two biological replicates at time points similar to the ones described earlier [18]. Erythromycin was quantified by LCMS as described in [21].

### RNA-sequencing

Deep sequencing was performed as described earlier [18], briefly, DSN treatment [32] and MicrobExpress (Ambion) were used for mRNA enrichment. Small RNA sequencing was performed after gel extraction and purification of RNA bands between 15 and 50nt. Illumina small RNA sequencing protocol was used for sequencing with minor modifications as previously described [18]. Total RNA was extracted using two cycles of cellular lysis in RNase-free zirconia beads, followed by column purifications. RNA quality was evaluated using BioAnalyzer (Agilent) and Nanodrop 1000 (Thermo Scientific) prior analysis. Ribosomal RNA was removed with MicrobExpress Bacterial mRNA Enrichment kit (Ambion) or duplex-specific thermostable nuclease enzyme from Kamchatka crab (DSN). Small RNA sequencing was performed by sequencing fragments of 15-50nt fractions excised for from a PAGE gel and purified for sequencing using the Illumina small RNA sequencing protocol as previously described [33]. All sequencing was performed at Geneworks (Adelaide, Australia) on the Illumina GAII. RNA sequencing data is available at GEO GSE39722 and on a dedicated *S. erythraea* genome browser http://pathway.aibn.uq.edu.au/serythraea.

### Proteomics

Proteins were extracted from cell pellets sampled at six time points of the fermentation as described in [34]. Cells were lysed using glass beads for 5 minutes at 4800 rpm. Two mg of digested proteins were digested overnight with Trypsin (Promega) and analysed via 2D-Nano-LC MS/MS. The first LC dimension was conducted offline on an Agilent 1200 HPLC, using a 1 mL strong-cation-exchange Resource S column where 16 fractions were collected [34]. The mass spectrometer, QSTAR-Elite (ABSciex), was equipped with a nano-spray ESI sources operated in positive ion mode coupled to a Nano-LC (Shimadzu Prominence). Peptides were separated using a flow rate of 30 μl/min on a Vydac Everest C18 column (300 A, 5 μm, 150 mm × 150 μm) at a flow rate of 1 μl/min and a gradient of 10-60% mobile phase B over 90 min. Analyst® Software (version 1.5.2, AB Sciex) was used for peak picking with a method searched for masses of 300 to 1800 Da. Information Dependent Acquisition (IDA) selected for +2 to +4 charges which exceeded 150 counts using Enhanced Resolution scans. The two most abundant ions in each of these scans (or with unknown charge) were subjected to MS/MS. An Enhanced Product Ion scan was used to collate fragment ions and present the product ion spectrum for subsequent database analysis. Protein Pilot Software v 4.0 (Applied Biosystems) and the Paragon Algorithm [35] were used for peptide identification using a fasta-formatted file with all protein sequences reported for the *S. erythraea* genome in NCBI and all the intergenic regions translated in six frames using BioJava 3. The theoretical ions and peaks were matched using the tolerance used by the Paragon Algorithm search, based on information about the mass accuracy of the instrument chosen in the Paragon Method dialog box. Search parameters included iodoacetamide as cysteine modification, trypsin as enzyme for protein digestion and 'Thorough ID’ search effort using a detected protein threshold of 95% allowing for false discovery rate analysis (FDR). Only proteins with a confidence score of 95% or better (estimated global FDR 5% or lower) were accepted. For a protein to be identified, at least two 95% confident independent peptide identification were required.

### Bioinformatics tools

KEGG SSDB [36] (sequence similarity database) and SMART [37] (Simple Modular Architecture Research Tool) were used to search for protein domains. String [38] (Search Tool for the Retrieval of Interacting Genes/Proteins) database was used to analyse genomic context and enzyme occurrence.

The genome annotation pipeline uses Prodigal 2 for gene finding [9]. We first assigned primary annotation by matched *S. erythraea*’s genome sequence against Swissprot and Interpro or closely related, well annotated microorganisms (including *S. coelicolor*, *S. lividans*, *S. avermitilis*, *Mycobacterium tuberculosis*, *Corynebacterium glutamicum*, *Frankia sp* and *Rhodococcus equi*). UniProtKB/Swiss-Prot databases [39] were further used to assign protein domains for all the sequences with no hits. Go and InterPro IDs were assigned using InterProScan [40,41]. tRNA and rRNA identification was performed using tRNAscan-SE [42] and rRNA_hmm_fs (Ergatis) respectively. All genes annotated as hypothetical proteins were analysed using InterproScan [43] to assign GO/InterPro IDs. For proteogenomics, all the analysis was done as described elsewhere [44]. Pinstripe is available for download at (pinstripe.matticklab.com). RNA sequencing analysis and read alignment were done as described in [18]. Briefly, reads were aligned using Bowtie2 requiring no more than 2 mismatches. Gene expression was normalized within libraries and between libraries as indicated in [18]. Statistical tests and figure generation were conducted using the Prism 5 (http://www.graphpad.com/prism/). For sRNA analysis and RBS analysis SAMtools [45] and additional in-house perl scripts provided at http://matticklab.com/index.php?title=Marcel_Dinger were employed.

## Competing interests

The authors declare no competing financial interests.

## Authors’ contributions

EM, CLC, TRM and LKN designed the experiments. EM and CLC performed all fermentation and extractions. EM and CLC performed protein purification and analysis. EM, RWP and TRM performed all bioinformatics work. EM, CLC and LKN wrote the manuscript. All authors read and approved the final manuscript.

## Supplementary Material

Additional file 1: Table S1 Comparison between the *S. erythraea* original genome annotation and the annotation performed with Prodigal2. **Table S2.** Novel genes identified. Unique identifier, chromosome location and highest BLAST hit indicated. **Table S3.** Proteins identified by nano-2D LC-MS/MS. Gene identifier and presence in sampled time-point indicated. **Table S4.** List of previously annotated hypothetical proteins detected by nano-2D LC-MS/MS. **Table S5.** Novel genes validated by nano-2D LC-MS/MS. Identifying peptide found by LCMS indicated. **Table S6.** Functional annotation suggested using the *S. erythraea* GSMR. Proteomics raw data, PRIMP analysis and the gene bank file are available online at http://pathway.aibn.uq.edu.au/serythraea/index.html. RNA-seq data is available from GEO GSE39722. Click here for file
